# Support of mathematical thinking through embodied cognition: Nondigital and digital approaches

**DOI:** 10.1186/s41235-017-0053-8

**Published:** 2017-02-20

**Authors:** Cathy Tran, Brandon Smith, Martin Buschkuehl

**Affiliations:** MIND Research Institute, Irvine, CA USA

**Keywords:** Mathematics education, Embodied cognition, Manipulatives, Gestures, Body movement

## Abstract

Research on mathematics education has shown that learners’ actions can influence how they think and vice versa. Much of this work has been rooted in the use of manipulatives, gestures, and body movements. Our article dissects the mechanisms that underscore the impact of embodied activities and applies this lens to explore how to harness the affordances of new technology to enhance mathematical thinking. This is especially crucial given the increasing accessibility of technology—such as digital touch devices, 3D printers, and location sensors—for constructing embodied experiences. Providing guidance for incorporating those tools, we focus on the role that embodied cognition can play in communicating mathematical concepts as well as in allowing learners to experiment and evolve their ideas. To inspire future integration of theory in the development of technologically enhanced embodied mathematics experiences, we provide examples of how this can be done. Finally, we outline future directions in the areas of design, implementation, and assessment of embodied learning of mathematics.

## Significance

Emerging technology offers novel ways to teach mathematical concepts. We explore these new opportunities for education and start by leveraging decades of research on embodied cognition that have given us insight on how people learn. Such research provides insight about the foundation of human perception and how learners process and integrate knowledge, elements that are highly relevant to any educational experience, digital or not. Our paper introduces the embodied cognition research in the context of manipulatives, hand gestures, and whole-body movement so that educators and scientists can leverage that foundational knowledge to further research and develop technologically enhanced embodied mathematical activities.

## Introduction

Embodied cognition refers to the idea that features of human cognition are formed not only by our brains but also by other aspects of our bodies (Wilson, 2002). The relation between embodied cognition and learning has a long history with decades of research that have influenced classroom curricula across domains including science, linguistics, and mathematics (Chan & Black, 2006; Glenberg, Gutierrez, Levin, Japuntich, & Kaschak, 2004; Lakoff & Núñez, 2000). The implementation of embodiment in these domains is evolving as novel interfaces and technologies are increasingly surrounding us, allowing for direct manipulation and immersive experiences through tools including touch devices, motion sensors, and virtual reality. In this article we argue that these technologies may provide largely untapped potential to increase learning effectiveness via an embodied cognition approach. We focus on the subject of mathematics because adequate mathematical knowledge is increasingly important in our technology-based society and young children’s mathematical development is an important predictor of their later academic achievement and labor market success (Ritchie & Bates, 2013). Further, mathematics naturally connects human perception and action, and therefore embodiment in this domain seems especially helpful in understanding its abstract, complex nature.

As we explore the new opportunities for education that these technologies allow, it is a sensible starting point to leverage decades of research on embodied cognition that have given us a window into how people learn. From an embodied cognition perspective, we will first discuss previous research in the context of manipulatives, hand gestures, and whole-body movement because those areas have shown promise of learning effectiveness in mathematics education and have been popular topics of research. Based on this background, we present technologically enhanced examples of embodied mathematical activities. Finally, we end with recommendations for future research and development directions related to embodied cognition, technology, and mathematics learning.

## Review

### Embodied cognition defined

Embodied cognition is a decades-long branch of research that encompasses a diverse set of theories that are based on the idea that human cognition is rooted in the bidirectional perceptual and physical interactions of the body with the world (Gibson, 2014; Wilson, 2002). Ways of thinking, such as representations of knowledge and methods of organizing and expressing information, are influenced by the perceptual and motor systems including body shape and movement, neural systems engaged in action planning, and systems involved in sensation and perception (Glenberg, 2010). Embodied cognition implicates a perception-action cycle in which behavior consists of a succession of adaptive motor reactions to changes in external (e.g., moveable objects) and internal (e.g., motivation) environments (Fuster, 2003). These actions (e.g., body movements) produce changes in those environments that in turn affect subsequent actions, continuing as a circular process through the central nervous system as sensory or internal signals lead to actions that generate feedback that regulates further actions.

The perception-action cycle that underlies embodied cognition also holds true for visual and symbolic representations of actions. For example, mental imagery is used to understand the positions of three-dimensional objects after rotations (e.g., Shepard & Metzler, 1971) and actions are simulated during language comprehension (e.g., Glenberg & Kaschak, 2002). As we further detail in our article, gestures are also an outgrowth of simulated action and perception. While the hand movements are clearly actions, they do not have a direct effect on the world and are instead representational. Further, the embodied cognition view that we adopt also extends the perception-action cycle to neural representations that connect previous actions with one’s thinking. According to the perceptual symbol systems (Barsalou, 1999), one’s neural representations of events are based on brain states that were active in the past during the actual perception and interaction with the objects and events in the real world. Perceptual symbols are believed to be multimodal traces of neural activity that contain at least some of the motor information present during actual sensorimotor experience (Barsalou, 1999). To give a concrete example, in related research with individuals who are skilled versus nonskilled in motor activities such as dance, skilled ballet dancers who viewed dance videos showed greater activations of brain regions that support motor actions than those who were not skilled in ballet (Calvo-Merino, Glaser, Grezes, Passingham, & Haggard, 2005). These neural representations resulting from physical interactions can represent a form of “offline” embodied cognition that could transfer the learning gained from physical actions to nonphysical tasks.

### Embodied cognition and learning

#### Levels of processing

Research in education has shown that bodily movements improve retention of the learned concept by providing additional cues with which to represent and retrieve knowledge (Carbonneau, Marley, & Selig, 2013; Chu & Kita, 2011; Lindgren, 2015). Taking action in response to information, in addition to simply seeing or hearing it, allows for an integration of modalities for deeper levels of processing to create a stronger memory trace that allows learners to activate multiple avenues for recalling the memory later on (Craik & Lockhart, 1972). Memories from movement can prepare learners for future action and can be retrieved to solve related tasks in different situations that no longer engage physical movement, but rather, a mental transformation of those motor processes.

#### Cognitive load

Movement can allow learners to reduce their brain’s processing power or cognitive load, leaving more resources for other activities or cognitive processes, which can result in improvements of problem-solving abilities (Sweller, 1988). For example, instead of trying to imagine how an object would appear when rotated, learners can reduce this burden of tracking information by allowing their hands to do it and seeing what happens. Freeing up those mental resources can allow them to think more deeply about spatial relations and have a better understanding of that concept before transitioning to spatial reasoning more abstractly. In a similar vein, gestures during explanations of math problems also help learners to track their thinking and reduce working memory usage so that they can allocate more cognitive effort to problem solving (Goldin-Meadow, Nusbaum, Kelly, & Wagner, 2001).

#### Connecting the abstract and the concrete

Physical movements complement humans’ natural tendency for learning because before abstract forms of thoughts such as mathematics emerged, problem solving in the real world required moving through space and manipulating actual objects (Wilson, 2002). This natural desire to situate cognition with real contexts is reflected in the mind-body connections of mathematical concepts such as embodied numerosity (Moeller et al., 2012) and the use of temporal and spatial metaphors (e.g., “the number is approaching zero”). Integrating the body into the learning experience can, therefore, improve mathematical understanding by providing a connection between concrete referents and abstract concepts.

### Embodied cognition and mathematics understanding

There is ample evidence that different aspects of mathematics are embodied. A prime example is the use of fingers to count and solve arithmetic problems (Fischer & Brugger, 2011). It is quite common to observe that especially young children use their fingers in combination with mathematical tasks. Such an observation is not surprising given the fact that fingers are typically readily available and cover the number range within which children are usually introduced to counting and arithmetic. This usage of fingers is a manifestation of embodied cognition and in the case of counting has been labeled embodied numerosity (Moeller et al., 2012). The link between numerical representation and finger representation has been established in different studies and experiments. For example, one line of research has been investigating the relationship between finger gnosis and mathematical abilities. Finger gnosis is the ability to perceive and distinguish fingers of one’s own hands without visual guidance. Typical tests to assess finger gnosis require hiding the participant’s hand from view while the examiner is lightly touching one or more fingers. The participant then has to identify which finger or fingers were touched. Several studies find that better finger gnosis is related to higher levels of numerical competence (Fayol, Barrouillet, & Marinthe, 1998; Fischer, 2008; Newman, 2016; Noël, 2005; Penner-Wilger et al., 2007; Poltz, Wyschkon, Höse, von Aster, & Esser, 2015; Wyschkon, Poltz, Höse, von Aster, & Esser, 2015). However, there is a set of newer studies that demonstrate that even though finger gnosis is a significant predictor of later mathematical competence, its practical importance as a predictor is nonexistent as it explains only about 2–4% of variance in 5.5–6.5-year-old children (Wasner, Nuerk, Martignon, Roesch, & Moeller, 2016; Wyschkon et al., 2015).

The use of fingers is also an impressive example to illustrate how physical body features influence how individuals process numbers. Specifically, as a consequence of finger counting, a sub-base 5 system seems to develop in western cultures (Domahs, Moeller, Huber, Willmes, & Nuerk, 2010). A sub-base 5 system manifests itself in the representation of numbers that are larger than 5, as 5 + another number. For example, the number 8 is represented as 5 + 3 and not 4 + 4; in other words when the number 8 is presented with fingers, five digits on one hand and three digits on the other hand are shown. Somewhat related, it has been shown that finger representations get internalized especially in the first years of schooling. This led to the observations of more split-5 errors than expected by chance (Domahs, Krinzinger, & Willmes, 2008). Split-5 errors describe errors in addition and subtraction problems that are deviating by +5 or −5 from the correct solution. These errors originate in situations where sums are larger than 10. In these situations, a two-digit result must somehow be represented with only 10 fingers. In order to accomplish this, a full hand must be reused and a failure to keep track of reused full hands results in a specific error, called a split-5 error. Such strong sub-base 5 effects have not been found in adults which seems to indicate that the relationship of finger and number representation is stronger in early elementary school and then diminishes from that point on (Domahs et al., 2008). This notion is further supported by a longitudinal study that investigated the correlation between the frequency of finger use and the accuracy of addition and subtraction problems (Jordan, Kaplan, Ramineni, & Locuniak, 2008). The study tracked children starting in kindergarten until the end of second grade and found that initial correlations were *r* = 0.60 and declined to *r* = −0.15 at the last measurement point. The results of this study indicate that finger use initially provides a natural scaffolding structure for calculation but then the benefits of using fingers fade, likely because fingers do not effectively deal with the complexity of later mathematics.

What can be learned from this literature is how interlinked at least parts of our bodies and thought processes are. But it is also clear that this relationship is not a simple one and varies across the lifespan (see also Newman, 2016). There are many other examples that further illustrate embodied cognition, beyond finger usage, in the domain of mathematics. Related to the SNARC (Spatial-Numerical Association of Response Codes)^1^ effect (Dehaene et al., 1993) are the findings that individuals produce smaller random numbers if their heads or bodies turned to the left and larger numbers if their head or bodies turned to the right (Loetscher, Schwarz, Schubiger, & Brugger, 2008; Shaki & Fischer, 2014). Further examples can be found in the gestures literature, the manipulatives literature, and the whole-body movement literature. A more detailed discussion of these domains and their relationship to embodied cognition is provided in detail in later sections of this article.

### Emerging technologies: implications for embodied cognition

Technology is increasingly allowing learners to have a greater degree of direct interaction with their digital environments, to include bodily movement in their interactions, and to be more immersed in those contexts. Touchscreens, for instance, provide affordances for direct tapping, sliding, pinching, and rotating gestures. This allows for an increased degree of gestural congruency such that physical movements correctly simulate cognitive processes (Segal, 2011). Building on that, sensors, such as the commercially available Nintendo Wii Remote and digital dance mats, allow for the tracking of arm and leg movements, allowing for increased motoric engagement. Technological advances of Microsoft’s Xbox Kinect and Leap Motion allow full-body motion detection, adding to the ability to incorporate natural interactions within the digital learning environment. The large displays that are often connected to those devices also allow for an increased perception of immersion. Virtual reality, such as through Google Cardboard viewers or by means of cameras projecting virtual content onto real-world environments (augmented reality), also add layers of context and depth to create more immersive environments. These types of technology provide opportunities for incorporating a greater level of embodiment into mathematical learning.

**Fig. 1:**
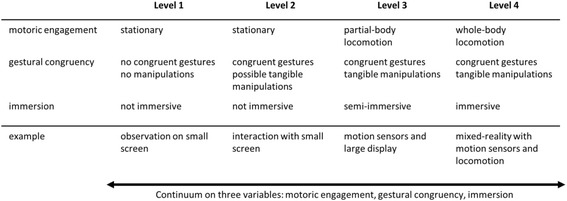
Taxonomy for the degree of embodiment in technology after Johnson-Glenberg et al. (2014). Embodiment can vary along three dimensions: motoric engagement, gestrual congruency, and immersion. Although it can be assumed that the three dimension follow a continuum, it makes practical sense to reduce the resulting complex three-dimensional space into categorical units that are labeled level 1 through level 4. Level 1 is assumed to represent the smallest degree of embodiment and level 4 the largest degree of embodiment

To identify the level of embodiment, Johnson-Glenberg, Birchfield, Tolentino, and Koziupa (2014) suggested a taxonomy which can be represented through the following three components that we already described above: (1) motoric engagement, (2) gestural congruency, and (3) immersion. It is helpful to build upon this taxonomy in thinking about the use of technologies in enhancing embodied learning experiences for mathematics. These three components vary along continuous dimensions which together define the degree of embodiment. However, given that the resulting three-dimensional space would be too complex in order to be practically useful, Johnson-Glenberg et al. (2014) proposed to reduce this space into categorical units. In Fig. 1 we outline four levels of embodiment following the example of Johnson-Glenberg et al. (2014), with level 1 representing the lowest degree of embodiment which is defined through minimal motoric engagement, no gestural congruency, and a nonimmersive experience. In contrast, level 4 represents the highest degree of embodiment through whole-body movement, gestural congruency and tangible manipulatives, and a highly immersive experience. Between these two extreme endpoints are two further levels which represent moderate degrees of embodiment.

Though the bulk of these technologies had been developed decades ago, it is only more recently that they have entered the mainstream, becoming accessible to students and educators to integrate into educational experiences. There is no guarantee, however, that emerging technologies will cue the kinds of body movements that have shown promise for effectively teaching mathematics. This leaves it up to researchers, designers, and educators to make deliberate efforts to implement novel technologies in ways that trigger movements that support, rather than hinder, targeted learning outcomes. We hope to further advance this conversation and provide insights for the research and development of embodied learning experiences. In the following sections we review the available literature about embodied cognition and mathematics in the domains of manipulatives, hand gestures, and whole-body movements. Additionally, we discuss how technology can be leveraged to enhance mathematical learning experience through embodied cognition and discuss related design opportunities. Finally, we will discuss future directions in the areas of design, implementation, and assessment of embodied learning of mathematics.

## Manipulatives

### Theoretical background

Manipulatives are objects that students interact with to learn, and these objects could be concrete or digital as long as students can slide, flip, and turn the visual representation as if it were a real three-dimensional object (Moyer, Bolyard, & Spikell, 2002). Several complementary theoretical assumptions provide insight as to how manipulatives impact learning. First is the thought that manipulatives support the development of abstract reasoning for younger children who have greater dependency on physically interacting with their environment to extract meaning (Montessori, 1964; Piaget, 1962). Those who are too young, however, may struggle with the concept that an object can stand for an item while simultaneously representing a mathematical concept (Uttal, O’Doherty, Newland, Hand, & DeLoache, 2009). Supporting this notion, a recent meta-analysis showed that children ages 7–11 years benefitted most from concrete manipulatives whereas ages 3–6 years found little benefits from using manipulatives. Concrete manipulatives have also been found to be less beneficial for older students, a finding that can be partly explained by their increased ability to reason abstractly (Carbonneau et al., 2013).

A second theory is that manipulatives provide the learner with an opportunity to enact the concept for improved encoding. In particular, the levels of processing notion (Craik & Lockhart, 1972) supports that teaching information in multiple ways, such as visually and symbolically, results in learners being able to activate those different modes when retrieving the knowledge. In the context of manipulatives, in learning how to solve an addition problem of 5 + 7 with blocks, students are able to code their understanding both with the symbolic code of 5 + 7 as well as the visual and kinesthetic code of interactions with the blocks. When later asked to do similar addition problems, students have access to multiple codes and the retrieval of one could activate the other, resulting in improved learning outcomes. This building, strengthening, and connecting of various representations of mathematical ideas enhances mathematical understanding.

Finally, manipulatives work by affording opportunities for learners to discover mathematical concepts through their own exploration. Generating an answer compared to just reading it has large positive effects on long-term retention of that material, an effect known as the generation effect (Bertsch, Pesta, Wiscott, & McDaniel, 2007; Slamecka & Graf, 1978). These effects are thought to occur because the processes during generation of knowledge, compared with being given the knowledge, are in greater alignment with those used to produce answers during testing (for details on the related transfer-appropriate processing framework, refer to Morris, Bransford, and Franks (1977)). It is also cognitively more effortful to generate a solution by yourself, which in turn might induce more active processing and strengthen the knowledge in memory (Bertsch et al., 2007; Pyc & Rawson, 2009). Recent neuroscience research building on this work to provide more insight about the mechanisms at play have resulted in findings that during a testing session, students who learned through generating their own solutions rather than being given solutions to reproduce and practice not only performed about 10% better but also showed lower activation in their left angular gyrus and precentral cortex/Brodmann area 6 (Karlsson Wirebring et al., 2015). This supports the possibility that students who generated their own solution had an easier time accessing their memory of a solution method and needed to engage working memory processes at test to a relatively lower degree than those who did not generate their own solutions.

### Effectiveness of manipulatives

The question of whether or not manipulatives are effective though does not have a clear-cut answer. A recent meta-analysis of 55 studies (Carbonneau et al., 2013) showed that instruction that used manipulatives, compared with abstract symbolic instruction, produced a moderate- to large-sized effect when students were measured on retention and small effects when higher level outcomes, such as problem solving, transfer, and justification, were considered. An analysis of moderators showed that a host of factors mattered such as age and the associated developmental status of the child, perceptual richness of the material, level of instructional guidance, and mathematical topic. Specifically, studies conducted with children aged 7–11 years old resulted in higher effect sizes (*d* = 0.81) than studies with children 3–6 years old (*d* = −0.09) or children older than 12 years (*d* = 0.31). Lower perceptual richness (e.g., plain blocks) yielded higher effect sizes (*d* = 0.77) than higher perceptual richness (e.g., toy pizzas; *d* = 0.28). Higher effect sizes were found for the mathematical topics of fractions (*d* = 0.93), algebra (*d* = 0.84), and place value (*d* = 0.70) than for geometry (*d* = 0.57) and arithmetic (*d* = 0.39). Finally, high instructional guidance (*d* = 0.90) for the manipulatives resulted in greater learning benefits than low instructional guidance (*d* = 0.19).

The amount of perceptual richness and the structure of the manipulative both have an effect on learners’ outcomes. Martin and Schwartz (2005), for example, taught children about fractions using either pie wedges or tiles. Findings showed that those who used tiles were better able to transfer their fraction addition skills to other manipulatives than those who used pie wedges. The hypothesized mechanism that underlies these findings is that the pie wedges’ structure already gave the learner a part-of-wholes-interpretation so they did not learn how to make and interpret such groupings and whole structures by themselves (Martin & Schwartz, 2005). Therefore, while pie wedges can initially help students with problem solving in that specific context, the added structure added perceptual richness that prevented them from being able to transfer that knowledge to other types of problems. In another study that looked at perceptual richness, fourth- and sixth-grade students were either given perceptually rich bills and coins (i.e., looking similarly to real-world bills and coins) or given bland bills and coins (e.g., white paper with black text indicating the numerical value) to help them solve problems involving money (McNeil, Uttal, Jarvin, & Sternberg, 2009). Those in the perceptually rich group made the most errors, findings that could be supported by the explanation that the presence of the perceptually rich bills and coins may have been disadvantageous because it is harder for children to use salient objects to represent abstract concepts (see also Uttal, Scudder, & DeLoache, 1997). There is a trade-off between the two types of manipulatives because younger students who have not learned the relevant school-based algorithms may need the help of bills and coins to solve the problems whereas older students who have more domain knowledge may not gain any benefit from perceptually rich bills and coins (McNeil et al., 2009).

The level of instructional guidance also has a significant impact on the benefits of the use of manipulatives (Carbonneau et al., 2013). A meta-analysis of 108 studies that compared different types of instructional approaches found that unassisted discovery resulted in poor learning outcomes relative to providing instructional support (Alfieri, Brooks, Aldrich, & Tenenbaum, 2011), findings that align with an earlier review that indicated that guided discovery was more effective than pure discovery in helping students learn and transfer (Mayer, 2004). In particular, findings suggest that unassisted discovery does not benefit learners, whereas feedback, worked examples, scaffolding, and elicited explanations do (Alfieri et al., 2011). Explanations for this include the possibilities that more guided tasks lower the demands on working memory and executive functioning abilities to allow learners to direct those efforts to problem-solving processes (Kirschner, Sweller, & Clark, 2006).

### Leveraging technology for improving embodied learning using manipulatives

#### Connect the concrete with the abstract through better scaffolding of knowledge

Digital manipulatives can allow students to make the connection between concrete objects and more abstract objects to gain a better symbolic, conceptual understanding of the mathematical concept. A challenge of the concreteness and perceptual richness of some physical manipulatives, however, is that it makes it difficult to transfer knowledge and generalize to other contexts. Students may not recognize, for example, that a circle with one fifth shaded, the decimal representation of 0.2, and the fraction representation 1/5 all represent the same mathematical value. It is possible that physical, concrete manipulatives are better to learn with at the beginning, but that digital manipulatives are better for transferability. So, if learners can transition from concrete to digital, they may get the benefit of both. As a scaffolding technique, younger students can start with concrete manipulatives before using digital ones. Our later case illustration in this manipulatives section illustrates one way this could be approached.

#### Constrain options of what can be moved to isolate variables

The ability to control which elements learners can and cannot move can be leveraged to design more effective embodied learning experiences. As an example of this, we turn to the possibility of constraining options of what can be moved in ways that encourage learners to use different strategies. Related to this, a study by Manches, O’Malley, and Benford (2010) sought to test whether differences in manipulation behaviors predicted 5-to-7-year-old children’s problem-solving strategies in a numerical partitioning task. The children were asked to provide all the ways that a certain amount can be combined (e.g., the number of ways that 9 can be recombined are 9 and 0, 0 and 9, 8 and 1, 7 and 2, and so forth). Researchers found that children provided significantly more unique solutions in the manipulative condition with small plastic blocks as opposed to the condition with paper and a writing instrument. These differences could be explained by the affordance of the block condition which allowed the children to move multiple blocks at a time which was not possible in the paper condition. This has implications for strategy use and mathematical understanding because, for example, reversing combinations (e.g., 5 and 2 into 2 and 5) is much easier to perform when manipulating multiple objects at once than by subsequently manipulating one object after another. As a digital application of this, students could be first constrained to moving one block at a time on a touchscreen and then be given the opportunity to move two blocks at a time to develop their strategies and mathematical understanding.

#### Use emerging technologies to create physical manipulations on demand

The use of technology for educational purposes often induces a mental image of students interacting directly with digital devices. Viewing technology as a general tool, however, allows us to put it to work in different ways. The power of 3D printing technology, for instance, can be leveraged to create a customized and personalized manipulative that is not available or feasible to get otherwise. Manipulatives are generally associated with early learning to help students to formalize some of the early school mathematics ideas. But, with the emergence of 3D printing and their digital design platforms, one could also use technology to create nonstandard manipulatives to convey more complex mathematical concepts beyond the early learning that the vast majority of manipulatives typically target (Carbonneau et al., 2013).

**Fig. 2:**
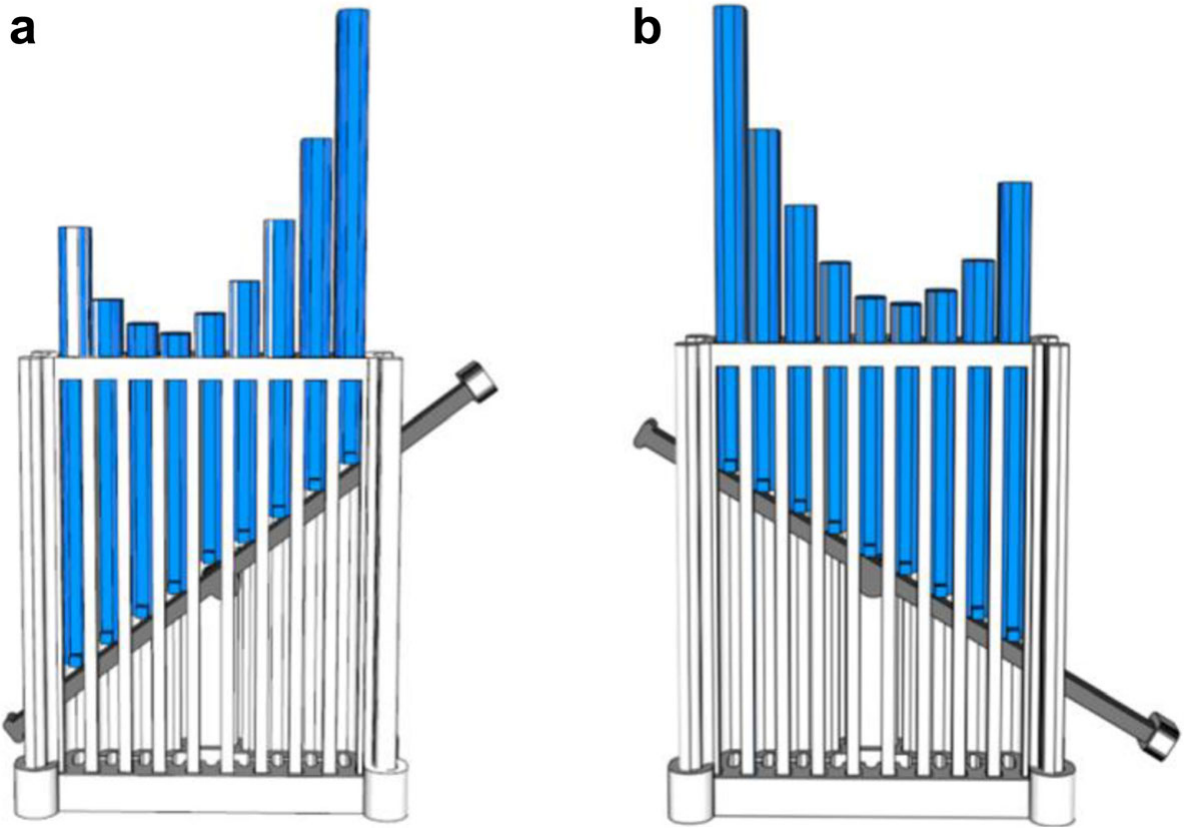
Panel **a** shows a parabola with a positive *b*-value; Panel **b** shows a parabola with a negative *b*-value

The manipulative shown in Fig. 2, for example, was designed as an on-grade-level tactile manipulative for high school mathematics.

Originally designed specifically for commercially available consumer 3D printers, this manipulative is intended for learning to graph quadratic functions. Commonly written in the form *y* = *ax* ^*2*^ + *bx* + *c*, students generally study the effects of changing *a* or *c*. However, it can be challenging to understand how the linear coefficient (*b*-value) influences the graph of a parabola. Because of this, students are not exposed to how this coefficient behaves in a graphing context. This manipulative allows students to change the *b*-value and see how it affects the graph of a parabola. A video of this manipulative in action can be viewed at https://www.youtube.com/watch?v=PmrzcR4H6xw.

#### Understanding very small or very large temporal and spatial dimensions

**Fig. 3:**
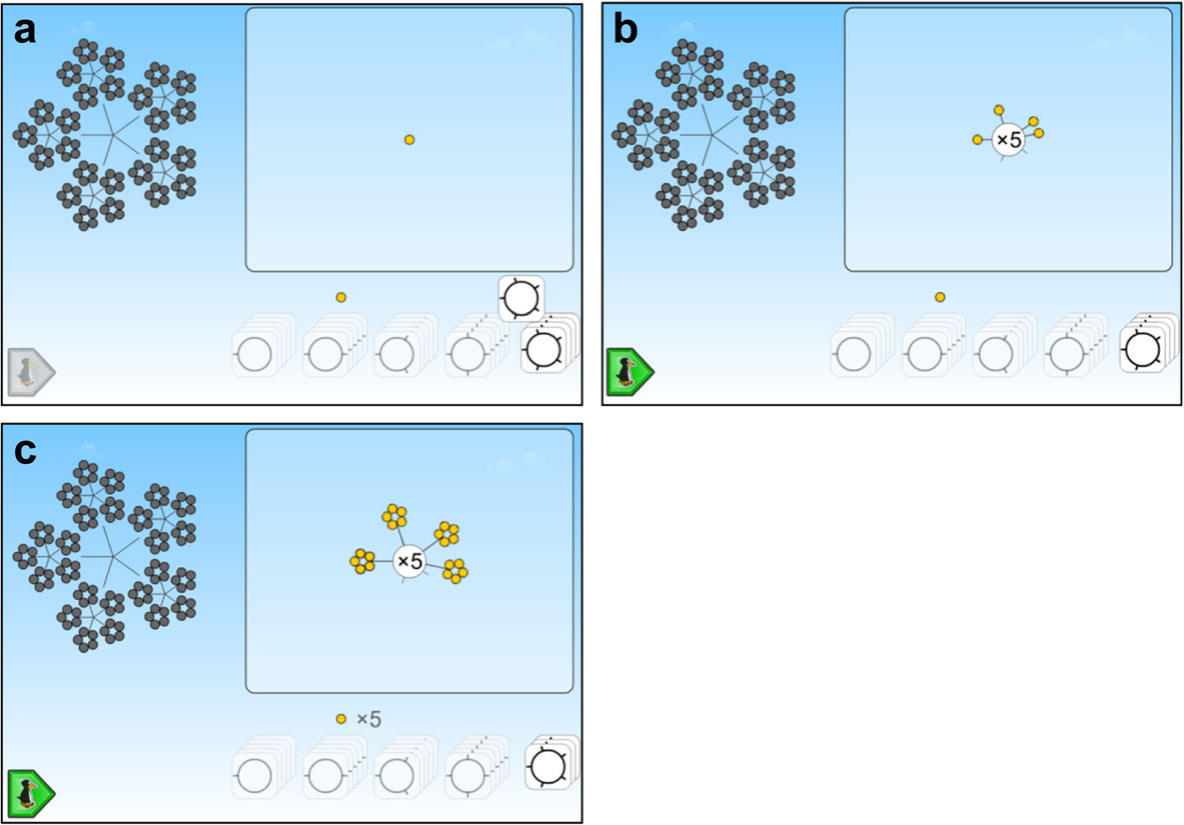
Panel **a** shows the screen resulting from a student selecting which card to apply to the circle in the large rectangle. Panel **b** shows the effect of applying the card to start generating the pattern on the left. Panel **c** shows the multiplicative effect of repeated use of the card

Digital manipulatives allow for understanding very small or very large scales such as the mathematical concept of exponential growth. For instance, in a game called *Circle Exponents* (Fig. 3), students arrange circles into clusters and see them exponentially grow. The software can zoom out to allow students to keep track of an exponential growing pattern, something unfeasible in a tactile environment. Additionally, students are able to see the circles organized into patterns that reinforce the mathematical concepts.

**Fig. 4:**
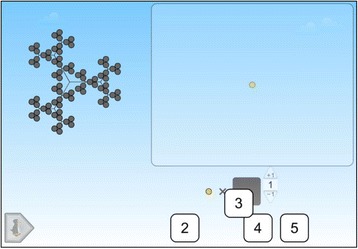
Students connect the pattern with exponential notation by writing an expression that models the pattern on the left

Students interact with the game by selecting a card with the correct number of spokes to replicate the yellow circle with the goal of creating the shape on the left (Fig. 3). The game moves from visual to symbolic as students describe the exponential pattern using visual cards, then repeated multiplication, then exponential notation (Fig. 4). By determining which card will replicate the circles, students are to create the graphic on the left. Students are asked to connect exponential notation with the graphic by generating the pattern and not just the total number of circles.

Though such a task can theoretically be accomplished with physical manipulatives, it would be logistically expensive and taxing, requiring the purchasing and space for spreading out thousands of objects in the classroom, ample time, and a great degree of patience from students. For further enhancing the embodied experience of exponential growth, one could conceive of virtual reality tools allowing students to feel exponential growth of objects all around them.

## A case illustration of Gyro JiJi

As a case illustration, we exemplify a way that technology can be used to connect the concrete with the abstract through better scaffolding of knowledge. One of the issues with learning with manipulatives is that if the manipulative is too concrete and too rich perceptually, students likely struggle to generalize that knowledge to other cases. However, especially for younger children, starting with an abstract example may be too challenging to grasp and learn. Technology has the potential to scaffold and link the concrete and abstract in ways that help students build those connections.

**Fig. 5:**
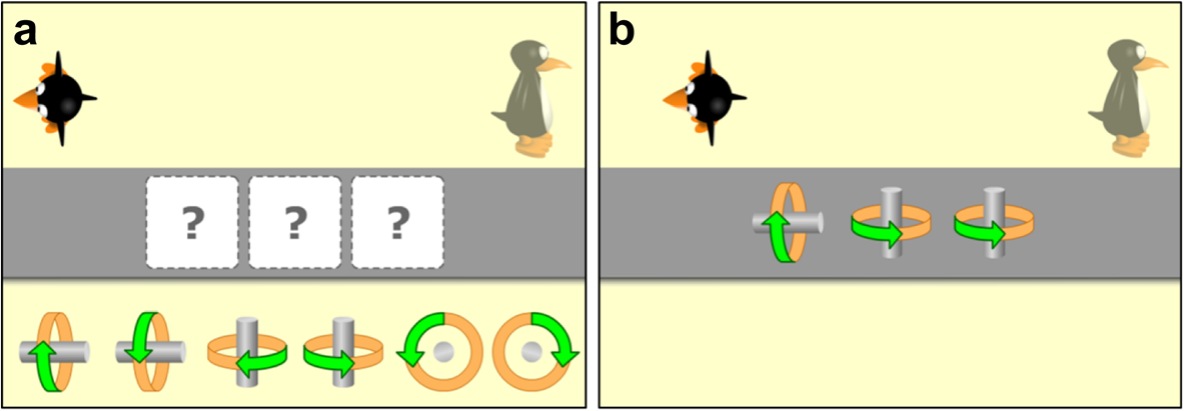
Panel **a** is a puzzle of Upright JiJi requiring three 90° rotations to orient the penguin in an upright position. Panel **b** depicts a solution that was entered before the penguin begins the selected rotations

As an example, we highlight a spatial thinking game that we enhanced through technology to provide a deeper embodied learning experience in reference to the embodiment taxonomy outlined earlier (Fig. 1) (Johnson-Glenberg et al., 2014). The game called Upright JiJi (Fig. 5) focuses on spatial-temporal reasoning in a three-dimensional state by allowing students to rotate a penguin (named JiJi) 90° along the x-, y-, and z-axes. The aim is to turn the penguin to an upright position by thinking ahead, selecting a series of rotations on a touch device, and then watching those answers unfold as the penguin rotates on the screen (Fig. 5).

**Fig. 6:**
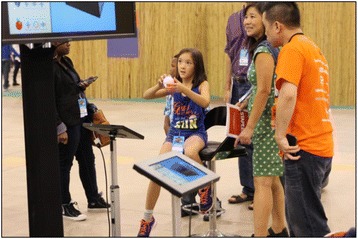
A student playing Gyro JiJi by rotating a gyroscope that is wirelessly connected to a computer that generates feedback on the screen

Leveraging technology to enhance this game, we developed an embodied experience for learners to connect with this relatively abstract concept through gestures by putting the penguin into the palms of the student’s hand to allow rotations to behave naturally as a real-time event. Rather than selecting the desired rotation and watching the animation, the learner is able to create the desired movement with a bluetooth-enabled gyroscopic device (Fig. 6).

As learners rotate the gyroscope in different ways, they interact with a combined physical and digital manipulative that translates their rotational hand movements into the rotations needed to rotate the penguin into an upright position.

## Hand gestures

### Theoretical background

Though gestures can take many forms, in this section we focus on hand gestures that often have been linked to mathematical thinking in the representative gestures literature (Alibali & Nathan, 2012) as well as the gestural congruency and interface design literature (Hostetter & Alibali, 2008; Segal, 2011). Representative gestures include *pointing* gestures to indicate objects or locations (e.g., pointing to a cube in order to refer to that cube), *iconic* gestures to illustrate concrete objects or actions (e.g., tracing a triangle in the air to mean *triangle*), and *metaphoric* gestures that resemble something concrete in order to represent something abstract (e.g., cupping hands as if to “hold” an idea). Gestural congruency refers to the alignment of the type of gestures used, such as on a touchscreen (e.g., tapping, sliding), with the mental model of the concept being taught.

Hand movements made with intention are often taken as evidence that the body is involved in thinking. Embodied cognition theories (Black, Segal, Vitale, & Fadjo, 2012; Wilson, 2002) purport that conceptual knowledge results in the activation of both perceptual and motor information, and thus, gestures influence learners’ construction of mental representations in mathematically relevant domains including spatial problem solving (Chu & Kita, 2011), mathematical equivalence (Perry, Breckinridge Church, & Goldin-Meadow, 1988), and counting (Alibali & DiRusso, 1999). The theoretical argument that gestures are linked with mathematical understanding is that those movements are reflective of a learning cycle in which perceptual encoding of math problems guide actions, and the consequences of those actions then guide what is perceived (Goldin-Meadow & Beilock, 2010; Hostetter & Alibali, 2008). This bidirectional, reciprocal relation between perception and action occurs because representative gestures, such as pointing to the two sides of a mathematical equation, can be explained by the learners’ perceptual encoding that there are two equivalent sides. Further, actions can also guide perceptional encoding. For instance, instructional gestures, such as asking students who are solving mental rotation problems to use their hands to represent how they would move the pieces to form certain shapes, changes their perceptions, resulting in better performance on the task (Goldin-Meadow & Beilock, 2010).

Gestures also impact learning through reducing cognitive load (Cook, Yip, & Goldin-Meadow, 2012; Goldin-Meadow et al., 2001). One mechanism by which this occurs, as demonstrated by a study that looked at memory and gesturing, is that gesturing while explaining math problems enables speakers to maintain more unrelated information in memory than they can when they do not gesture (Cook et al., 2012; Goldin-Meadow et al., 2001). This representational format is thought to free cognitive resources that can then be used to encode new information in a more lasting format.

### Effectiveness of gestures

Gestures have been shown to improve learning by helping learners process existing ideas with less cognitive load. Since hands are already commonly used to manipulate objects, gestures provide additional feedback and visual cues by simulating how an object would move if the hand were holding it. For example, gestures allow for the tracking of an object in the mind as it is getting mentally rotated, improving spatial visualization (Chu & Kita, 2011). In a series of experiments with allowed-to-gesture and prohibited-to-gesture groups of young adults, Chu and Kita (2011) found that people who have difficulty in solving spatial visualization problems spontaneously produce gestures to help them, and that the use of gestures is related to improved performance. As participants solved more problems, frequency in gestures decreased. It is thought that the spatial computation supported by gestures becomes internalized, and the gesture frequency decreases. This in part provides an explanation as to why the benefit of gestures persisted even in subsequent spatial visualization problems in which gesture was prohibited.

Gestures are also involved in creating and shaping new ideas by introducing new ways of thinking through movement. In one line of related work with elementary school students, one group was told to gesture while solving algebraic equivalence problems (e.g., 5 + 4 = 3 + 4 + ____) by putting their fingers in a “V” formation when referencing the addends of an equation whereas another group was not told to gesture (Broaders, Cook, Mitchell, & Goldin-Meadow, 2007). Findings showed that the gesture group solved more equivalence problems correctly in the post-instruction test compared to peers who were instructed not to gesture. In a similar vein, in a study of 4 and 5-year-olds who were taught about symmetry either through a lesson that included pointing gestures or a comparable one that did not, those in the gesture group correctly answered more than twice as many post-instruction questions when asked to judge six items as symmetrical or asymmetrical (Valenzeno, Alibali, & Klatzky, 2003). These studies point towards an untapped potential for prompting cued body movement to improve learning.

Gestures have been shown to be effective in helping learners to retain knowledge long term. Research demonstrates that learners should be more likely to grasp a concept if told to produce gestures instantiating that concept during learning than if told to verbally articulate the concept without using gestures. In a study with third- and fourth-grade students, three conditions for solving mathematical problems were compared: participants were either to produce gestures that displayed a strategy for solving the problem, to produce spoken words reflecting the same problem-solving strategy, or to do both (Cook, Mitchell, & Goldin-Meadow, 2008). Although all groups improved comparably, from pretest to immediate posttest, what is particularly interesting is that in a follow-up a month later, those in one of the conditions with gesture retained more knowledge than those in the speech-only condition. In explaining these results, the research team presented three hypotheses: (1) gesture offers a representational format that requires relatively little effort to produce, thereby freeing resources that can then be used to encode new information in a more lasting format, (2) gesturing directly facilitates encoding in long-term memory through producing stronger and more robust memory traces than expressing information in speech because of the larger motor movements involved or because of the potential for action-based, bodily encoding, and (3) gestures that indicate objects and locations in reality may make it easier for learners to link developing mental representations to relevant parts of the external environment to reduce processing demands.

### Leveraging technology to improve the benefits of gestures

#### Cue gestures that are congruent with the mental representations of the mathematical concept

The use of congruent gestures helps to construct better mental representations and mental operations to solve mathematical problems, which has implications for the ways that digital interfaces can be designed. Hostetter and Alibali’s (2008) Theory of Gestures as Simulated Action suggests that gestures emerge from perceptual and motor simulations that underlie embodied language and mental imagery. This suggests that asking learners to perform a specific type of gesture could mentally prime them to solve the problem in a particular way. For example, a study focused on children’s performance in arithmetic and numerical estimation provided interfaces for gestures that were congruent or incongruent for those mathematical models (Segal, Black, & Tversky, 2010). In particular arithmetic is a discrete task and should be supported by discrete rather than continuous actions whereas estimation is a continuous task and should be supported by continuous rather than discrete actions. If action supports cognition, performance should be better with a gestural interface designed such that the actions map conceptually to the desired cognition. As such, tapping with a finger on a virtual block or clicking with a mouse on a virtual block to count and add are gestures that are congruent with the discrete representation of counting. In contrast, sliding the finger vertically over a series of blocks or dragging a mouse across a series of blocks to count them are continuous movements that are not congruent with the discrete procedure of counting. In a 2 × 2 study with those four conditions, Segal and her colleagues (Segal et al., 2010) found that students who did arithmetic with a tapping gesture performed better than those who did it with a sliding gesture. Also as expected, students who did numerical estimation with a sliding gesture performed better than those who did the task with a tapping gesture. Later in this section we provide an example of applying this recommendation of cueing gestures that are congruent with the mental representations of the mathematical concept in the context of the mathematical understanding of slope.

#### Promote the development of effective strategies

In work that shows that teaching students gestures can help them to adopt new problem-solving strategies, as detailed earlier, Goldin-Meadow, Cook, and Mitchell (2009) used an abstract gesture to help 9- to 10-year-old children solve mathematical-equivalence problems such as 4 + 3 + 6 = __ + 6. Children were taught to spread out two fingers to make V-point gesture with the two fingertips each pointing to the first two numbers (the 4 and the 3 in this instance) and then have a finger on the other hand pointing at the blank on the other side of the equation. The purpose of these movements was to help the children see that the problems can be solved by grouping and adding the two numbers on the left side of the equation that do not appear on the right side and then putting the sum in the blank. Children who were asked to produce these hand movements during a math lesson were able to extract the grouping strategy despite the fact that they were never explicitly told what the movements represented. Future design and development work could explore ways to leverage technology to encourage gestures that contribute to effective strategy use.

## A case illustration of Tap Tempo

In this case illustration, we provide an example of leveraging technology to provide gestural congruency to provide students with another level of understanding of the concept of slope. Typical approaches teach slope as a formula to memorize and be applied to a static graph. Students look at two points and simply remember which to subtract and where to put the answers. Students have well-documented misconceptions when learning about slope of lines (Leinhardt, Zaslavsky, & Stein, 1990), resulting in them missing the connection between their calculations and the concept of a slope. Often, students learn the formula and practice calculating with it to the point that the formula abstracts away the understanding and hides what slope really means.

**Fig. 7:**
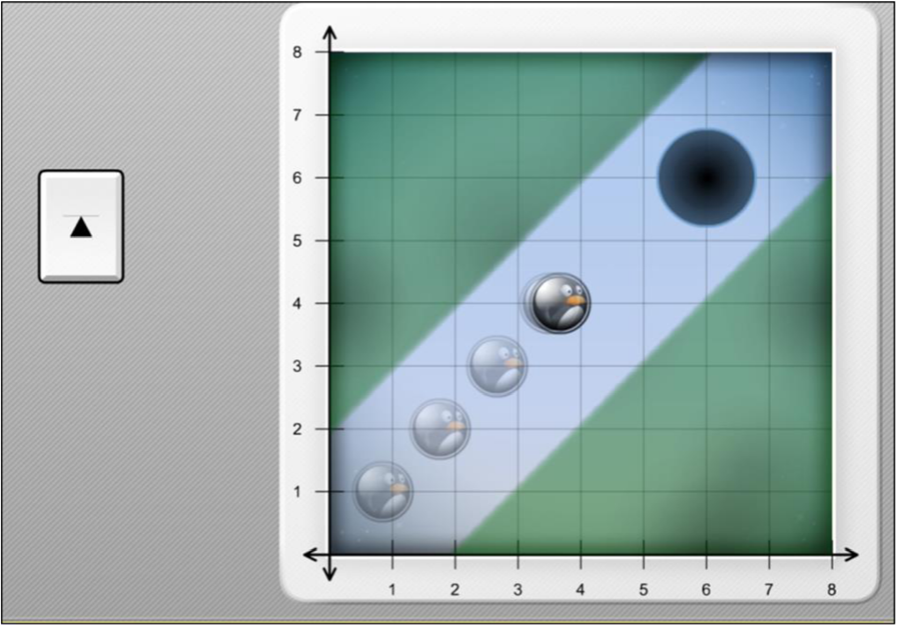
A puzzle of Tap Tempo that can be solved by tapping the button to move vertically over time

To address this issue, a game called Tap Tempo was designed to experience the difference in slope as a tapping tempo, providing the embodied experience of slope as a literal rate over time. Tapping influences the vertical movement such that faster tapping creates more steepness and, by having embodied control of this mathematical process, students can internalize that slope is about a vertical change over a specific horizontal distance (Fig. 7).

This allows students to experience the embodiment that a linear path has a constant slope and connect that with the understanding that a line is the only type of function for which a constant cadence along the x-axis corresponds to a constant cadence along the y-axis. As a form of gestural congruency in this game, lines with different slopes can be embodied with a faster or slower tapping tempo.

Further, another common error is that students subtract values inconsistently while calculating the slope, sometimes making errors with mixing up the order of y’s compared to x’s and sometimes computing subtraction of x’s against y’s and not getting slope at all. In a touch environment, the game can be designed such that the student controls both horizontal and vertical movement simultaneously. A slope of 1 means “same speed in horizontal and vertical.” A slope of 2 means you move your left hand (vertically) twice as fast as your right hand (horizontally). This affordance made possible by multitouch screens is not possible to implement with a computer mouse as input device.

## Whole-body movement

### Theoretical background

Another domain that has been repeatedly linked to embodied cognition in mathematics is full-body movement. Critically, by whole-body movement, we do not refer to exercise or workouts that strengthen muscles and the cardiovascular system, but instead focus on bodily activities that are closely related to mathematical content with a negligible fitness component. Similar to the domains of manipulatives and gestures that we covered before, the idea is that sensorimotor information, that is processed and accumulated during a body movement, facilitates mathematical learning (Fischer, Moeller, Bientzle, Cress, & Nuerk, 2011). The Theory of Event Coding (TEC) has been used as a theoretical foundation to explain how body movements as an embodied experience can impact learning (Hommel, 2009, 2015; Hommel, Müsseler, Aschersleben, & Prinz, 2001). This theory proposes, for example, that stored and strongly interlinked information about concrete objects in the world include not only perceptual features, such as color, shape, size, and smell, but also action-related properties such as chewing and swallowing. Critically, if an object is processed, all of its features – perceptual and action-related in nature – are activated. During later retrieval, the activation of one feature (i.e., color) then facilitates the activation of associated features (i.e., size). Moreover, the activated features (i.e., color and size) also facilitate the perception of other objects and actions that have similar features. But this may also work the other way around, that is, the activation of features of a planned action facilitates the perception of objects and carrying out other actions that share features with this planned action. Although TEC serves reasonably well as a theoretical starting point to explain embodied cognition, its original conception did not have embodied cognition in mind. However, that extension is straightforward, not only in regard to body movements, but in regard to embodied cognition in general (Hommel, 2015).

### Effectiveness of whole-body movement activities

Most of the currently available intervention studies on the impact of whole-body movements on mathematical learning focused on magnitude representation involving a number line. The main motivation to focus on magnitude representation in young children is the finding that early mathematical competence predicts future arithmetic performance (Booth & Siegler, 2008) and later achievement (Duncan et al., 2007). To our knowledge, there is currently only a limited set of intervention studies available that incorporated whole-body movement into the training part. Since this corpus of literature is so small, we are going to review the individual studies in more detail than we did with the literature in the previous sections where the available published research is considerably more substantial.

Fischer et al. (2011) conducted a study with the aim to improve number magnitude through an embodied intervention. They analyzed data of 5–6-year-old children who formed the experimental group and at the same time also served as a control group in a cross-over design. In the experimental condition, children had to decide whether a presented number was smaller or bigger than a presented target number using a digital dance mat. If the number was bigger than the target, they had to move their whole body by stepping to the right; otherwise they had to step to the left. With the goal to further increase the effectiveness of the intervention, a spatial number line was also presented in the experimental condition. In the control condition, the same task was performed on a computer with a touch interface without presenting a spatial number line. Identical number sets were used in both conditions. In each training condition, children trained for three sessions that lasted between 10 and 20 min. All six training sessions were held within 6 weeks. The authors reported that after the digital dance mat condition, children performed better in a traditional spatial number line estimation task and also in an untrained verbal counting task. No effects were found in object counting, knowledge of digits and number words, and simple addition and subtraction problems. We note that the design of this study does not allow for disentangling the potential effects of the dance mat component and the potential effects of the presentation of the number line, both of which were only present in the experimental but not the control condition.

The participants in the above described intervention study (Fischer et al., 2011) had to indicate the position of a number on a number line by making a categorical response on a digital dance mat. Since the number line is assumed to be continuous, however, it makes sense to implement a continuous oriented intervention, which is what Link, Moeller, Huber, Fischer, and Nuerk (2013) did. In their study, they trained first-grade students on a number line task that required participants to walk to a position on the line that corresponded to a given number. The response of the participants, that is, the position where they stood on the number line, was tracked with a Microsoft Xbox Kinect device. Again a cross-over design was operationalized in which all participants’ performance was tested in the experimental condition and a control condition. The control condition also involved a walking component that was not related to mathematical content such that participants were instructed to walk to a tablet computer and estimate the position of a given number on a virtual number line using a touch interface. This procedure ensured that neither the walking component nor the presentation of a number line represented confounding variables. The children were trained over three sessions in both conditions. The authors reported that both groups reliably improved in a number line task but that the improvements in the experimental and control conditions were not significantly different from each other. Neither were there effects found in symbolic and nonsymbolic number comparison tasks nor in tasks that measured place-value understanding. However, the authors found differential training effects in favor of the experimental group in two out of three measures of mental addition.

An issue of the two intervention studies reported so far is that they are not very scalable because the training sessions had to be conducted in a one-on-one setting. This inherently limits the potential reach of these embodied training approaches in the classroom. In order to overcome this problem, Fischer, Moeller, Huber, Cress, and Nuerk (2015) conducted a pilot study in which they used an interactive whiteboard as a means to train number magnitude in an embodied way. The authors argue that whiteboards are readily available in classrooms and are also big enough in order to implement embodied training of number magnitude with them. In their intervention, second-grade students had to mark given numbers on a number line drawn on a whiteboard. Critically, the whiteboard was big enough so that children had to walk a few steps in order to reach the position at which they marked the number on the number line. The whole intervention including pretest and posttest assessments was conducted in just one session. Two control conditions were implemented, one in which children had to discriminate colors on the whiteboard (media-matched control group) and another control condition in which participants performed a number line estimation task on a computer tablet (task-matched control group). The authors reported that the experimental group performed better in a number line estimation task than the two control groups but there were no other differential group effects in a mental addition task, a number comparison task, and a task in which children had to select the closest number to a given reference number.

In the study by Ruiter, Loyens, and Paas (2015), the authors compared two-digit number building training in an embodied condition with a control condition that did not involve mathematics-related whole-body movement. The first-grade students were instructed to build numbers using “blocks” of 10s, 5s, and 1s. So, for example the number 27 had to be built of 2 × 10, 1 × 5, and 2 × 1. In the embodied condition, the participants were instructed to build the numbers by taking steps along a ruler on the floor. Large steps corresponded to the 10s, medium steps to the 5s, and small steps to the 1s. In the nonembodied condition, participants had to build the numbers verbally, without taking steps, followed by indicating on a ruler where the number that they just built was located. After a brief intervention of constructing 10 numbers, results revealed that the embodied condition outperformed the nonembodied condition on two tasks that required building numbers using Lego blocks, in a similar way as the numbers were constructed during the intervention. It should be noted that this study implemented a posttest-only design, that is, participants were not tested on the criterion task before the intervention and, therefore, it is uncertain whether the groups were comparable at baseline.

Finally, in a recently published study, Dackermann, Fischer, Huber, Nuerk, and Moeller (2016) trained children to segment spatial distances into equal intervals with the hypothesis that a better understanding of the concept of equidistant spacing would lead to a better understanding of number magnitude. The theoretical motivation for this training approach is based on the observation that children frequently overestimate the position of small numbers on the number line such as by placing the number 10 where number 40 would be (Siegler & Booth, 2004). In a cross-over design, 22 second graders took part in the embodied training as well as the control training. In the embodied condition, children started to walk at the beginning of a number line that was taped to the floor and were instructed to choose their stride length so that it segmented the number line into a requested number of pieces. Every step defined one segment. Their steps were tracked by a Microsoft Xbox Kinect device which was necessary for performance measurement and generating performance feedback that was presented via video after each trial. In the control condition, children had to segment a number line shown on a tablet computer into equidistant pieces using a touch interface. It should be noted that the control condition also included a full-body movement component that was unrelated to any math activity, i.e., it involved walking to the tablet computer in order to segment the number line. The authors report that in the embodied condition children were better in segmenting a number line compared to the control condition. However, no condition differences were found for number line estimates and arithmetic performance.

The reviewed intervention literature that focuses on whole-body movement as an embodied way to improve numerosity is small and more work is needed to evaluate the potential of an embodied training approach in this domain. The current evidence of efficacy is based on relatively small sample sizes per study – with the exception of Ruiter et al. (2015) – and only one to three training sessions per condition. It is conceivable that longer interventions would result in more pronounced effects, assuming that more training leads to better learning. The reviewed studies demonstrate that participants got better in the condition of interest but this improvement was not always superior than the improvement in the control condition. Additionally, there is inconsistent support that participants also improve in tasks that were not part of the intervention such as verbal counting and mental arithmetic. With that it seems that other approaches, such as playing linear number games (Ramani, Jaeggi, Daubert, & Buschkuehl, 2017; Ramani & Siegler, 2008; Ramani, Siegler, & Hitti, 2012), are currently more effective as they not only lead to improvements in the trained task but also consistently result in improvements in untrained mathematics-related tasks. However, there are large procedural differences between the linear number line board game studies and the whole-body movement studies such as group versus one-on-one administration and number of training sessions to name only a few, that do not allow a straightforward comparison of the two intervention approaches. In sum, the available whole-body movement studies that aim to enhance mathematics learning are inconsistent and a clear advantage of the embodied approach is not yet fully established.

### Leveraging technology for designing whole-body movement activities

The basic idea of whole-body movement as an embodied activity is that it has a positive impact on cognition by allowing a student to learn a certain mathematical concept better than without movement. Here, we were only interested in movement that is closely related to mathematical content and not in movement for the sake of fitness or motivation. In the following we want to explore in what way we can use emerging technology as a superior tool to increase the efficiency of mathematical learning. It is worth mentioning at this point, that this area of research is relatively young and with the exception of one study (Ruiter et al., 2015) is already characterized through the involvement of a set of different modern technological devices such as the Microsoft Xbox Kinect, digital dance mats, and interactive whiteboards. However, as we also later discuss in more detail, there are other technologies that to the best of our knowledge have not yet been implemented in mathematical intervention studies, such as pedometers, Global Positioning System (GPS) trackers, and floor-projected imagery that changes as a function of where participants stand.

#### Whole-body movement as a thought process that can be reflected upon

Body movements can be tracked through technology. If such movements are executed to embody a mathematical principle then such tracking allows us to study the mathematical thinking processes that an individual is going through. As a consequence, we can present the learner with immediate, real-time feedback (e.g., Kim, Min, Kim, & Lee, 2014 for an example for yoga postures) or study the exhibited actions and underlying processes at a later point in time such as implemented by the reviewed study of Dackermann et al. (2016) who provided delayed feedback via video to their participants. The tracking of movements as an expression of mathematical thought processes is possible without interrupting the thought processes of a learner. In contrast, tracking thought processes in a more traditional setting often requires learners to deliberately put their own thinking into words by writing them down or saying them out loud as they are problem solving. The problems of the latter approach are that it is more intrusive and that a learner might fail to mention an important step in the train of thoughts or the description of thoughts is lacking detail. Further, real-time feedback on thought processes is very challenging to implement in traditional settings. On the other hand, one has to keep in mind that body movements are not a direct expression of thoughts; however, if implemented carefully, they might provide insights into ongoing thoughts that are not possible to access otherwise. Finally, recorded body movements can serve as an excellent source to reflect on the understanding of the mathematical problem at hand and allows for the discussion and comparison of mathematical solving approaches of different individuals.

## A case illustration of whole-body movement

An explicit goal of the following case illustration was to create an embodied activity that made efficient use of technology that would enhance learning without putting the technology itself in the focus of attention. In this activity, students first measured their average stride length and were then asked how far away they thought a certain object in the environment was, for example, a wall. Students were then instructed to give an estimate before pacing out the distance. Next, students walked the distance to the target and counted the number of steps. In order to improve their own measurement accuracy, they were asked to walk back to the point from where they initially started and calculate the average of their two measurements. While the students did that, a pedometer that was given to them beforehand, also measured their steps and allowed a comparison with their own measurement. In a next step, students could then calculate the distance by multiplying the counted number of steps with their average stride length. In order to get a more objective measurement of the real distance to the target, they were provided with a laser-based distance measurement tool which again allowed a comparison with their calculated distance. This activity was then repeated with different targets that were located in varying distances from the starting point. In this environment the main goal was to train number sense through practicing estimation skills, but other mathematical aspects, such as calculating averages and comparing expected numbers with real measurements, were also part of the exercise. Here, technology takes a more subtle role, and movement becomes a vehicle of mathematical intent. In this way, the power of technology is elevated due to its ability to facilitate without it being distracting.

In a somewhat related activity that is also on display in different variants in mathematics museums, such as the The National Museum of Mathematics (MoMath) in New York City (USA), students are asked to interpret functions of graphs through movement. A common approach is to define the x-axis as time. Often, graphs are perceived to be static, but there is mathematical foundation for them to be active. For example, one way to interpret the mathematical idea of parameterizing a curve, may it be linear or nonlinear, is acting it out over time. This creates an opportunity for students to act out functions while technological devices are visualizing the movement on a monitor; for example, through data acquisition of a pressure-sensitive floor mat. To interact with the technology, students stand on a mat several yards long. As they move forward or backwards on the mat, their position is interpreted in the context of the graph on the monitor. Walking forward on the platform, towards the monitor, is interpreted as movement upwards on the graph. Taking steps back on the platform models a vertical decrease. Visitors start at a fixed middle point and must step forward or backwards to stay as accurate to a given graph as possible. In such an environment, the slope of the graph becomes synonymous with speed along the y-axis. When a graph reaches a point where it becomes a horizontal line (slope = 0), individuals may interpret that as a temporary break where they can stand still. Obviously, environments such as this evoke a high degree of physical movement, and the movement is intentionally suggested by the graph.

## Discussion

We reviewed the influence of manipulatives, hand gestures, and whole-body movements on mathematical learning in the context of emerging technologies, and we discussed the potential to increase the impact that embodied interventions have in these domains. We find that the embodiment across all three reviewed domains can benefit mathematical learning, likely by providing an additional representation of the mathematical conception to strengthen encoding, by reducing cognitive load to provide more processing power to deeply think and problem solve, and by inspiring the use of strategies and modes of thinking other than what nonembodied approaches evoke. At the same time, however, it also becomes clear that the research on embodied cognition and mathematics is still quite young, in some domains more so than others. The available body of research on manipulatives as well as hand gestures is more comprehensive than the work on whole-body movement. Across the domains of manipulatives and whole body movements, most of the work in mathematics understanding has been conducted with younger children and, therefore, it is unclear what effects can be expected with an older population, such as college students, who are learning more sophisticated mathematics. Focusing on the research on the effectiveness of manipulatives, it seems that manipulatives are generally beneficial; however, results are strongly moderated by the contexts in which they are used and, in particular, the amount of instructional guidance and the perceptual richness of the material. Finally, it is an open question as to whether the different levels of embodiment, in the sense of the taxonomy suggested by Johnson-Glenberg et al. (2014; Fig. 1), have a corresponding impact on the learning success, so that level 1 embodiment would lead to less learning success than level-4 embodiment, with level-2 and -3 embodiment being between the two extremes.

In providing guidance for how we can leverage technology to design embodied experiences for mathematical understanding, the examples that we provided are intended to illustrate the possibilities and the constraints involved, but we acknowledge that there are other design approaches and technologies that can be used. For example, we did not discuss “social” technologies, such as collaborative work (e.g., Falloon, 2015; Plass et al., 2013), nor a more detailed analysis of augmented and virtual reality (e.g., Espejo-Trung, Elian, & Luz, 2015, for an example in dentistry). Future research and reviews certainly have much ground to cover in those areas, but there is still quite a bit that we can take away from and build on with what is currently known and what our reflections across three areas of embodied cognition and mathematics have revealed. The effectiveness of the case illustrations that we provided also has not been formally assessed which constitutes another necessary area for future exploration. In guiding the application of embodied cognition to the design of mathematical tools, we discuss thematic considerations for the design, implementation, and assessment in the space of technology, embodied cognition, and mathematics education.

### Guidance for designing optimal embodied experiences

#### Cue movements that align with the mental model of the mathematical concept

With the link between movement and thought, one of the opportunities that we have for design is that we can guide movements to influence thoughts in ways that improve mathematical cognition. This can be done on touch interfaces through cuing appropriate gestures, such as discussed earlier (see also Richland, 2015), tapping for discrete arithmetic operations, sliding for estimation problems, and tapping at different rates to experience different steepness levels of slope.

#### Make movements visible and give opportunities for reflection

A challenge to using body movements as a foundation or even as a metaphor for activating new learning opportunities is that individuals may not remember the details of the movements that they engaged in. For learners to reflect on their movements and the mathematical concepts that those movements enact, there needs to be a way for them to observe and reflect on these movements. To address this challenge, technology may allow a playback feature or provide real-time metrics that allow learners to observe what their body is doing with respect to the important features of the target learning domain.

#### Remember that less can be more

Technology is a tool. If treated as the focus, technology actually adds more distraction to the environment and may not result in better learning. Using technology does not have to be flashy and can be incorporated in subtle ways. This is what our case example does in which students are instructed to estimate distances and compare their estimates with their own real-world measurements using pedometers and laser-based measuring tools. Not only does the “less is more” adage help with cost, it also helps with learning. If the technology is too perceptually rich, it will distract from the learning content, as found in the case of certain manipulatives. Ideally, the technology blends perfectly into the activity, so that you are not blinded by it but you are able to fully focus on the content.

### Implementation opportunities and challenges

#### Logistical constraints and opportunities for scalability

Technology can help to make some things more scalable such as the use of manipulatives. When it is digital, one does not have to buy several copies of objects, set them up, and clean them up. The falling price of technology also helps with scaling technologically enhanced experiences. However, there are also aspects of embodied learning that are not as easily scalable. With whole-body movements, for instance, it is hardly feasible to have 20 or more digital dance mats or Microsoft Xbox Kinects in the classroom given the cost, required space, and supervision needed to implement this.

Further, 3D printing allows for the creating and sharing manipulatives using free software and open-source devices. As 3D printers become increasingly affordable and available in schools, custom manipulatives will become more scalable. Consequently, schools with 3D printers could receive digital files and print the manipulatives as needed. They could print in any size that suits their needs or even with the colors or materials that best fit their desires. This puts customized and personalized manipulatives into the classroom quickly and at low cost.

### Assessment considerations for measuring learning

#### Just because students are moving does not mean that they are learning

Learners may manipulate physical objects seemingly meaningfully, but that does not necessarily mean that the abstract concepts that these objects aim to convey are understood. In an example illustrating that, working with Cuisenaire rods, Holt (1982, pp. 138–139) said that he and his fellow teacher “were excited about the rods because we could see strong connections between the world of rods and the world of numbers. We therefore assumed that children, looking at the rods and doing things with them, could *see* how the world of numbers and numerical operations worked. The trouble with this theory is that [my colleague] and I *already* knew how the numbers worked. We could say, ‘Oh, the rods behaved just the way numbers do’. But, if we *hadn’t* known how numbers behaved, would looking at the rods enable us to find out? Maybe so, maybe not.”

#### Track not just learning outcomes but also readiness to learn

Research has shown that embodied learning can provide indicators of students’ readiness to learn. For example, in research on gestures, those who produce gesture-speech mismatches have been shown to be more ready to learn (Goldin-Meadow & Wagner, 2005). Their research suggest that what gestures provide is particularly useful when speakers are at the cognitive brink of learning something new because during those moments, gestures can reveal thoughts that learners cannot express verbally. Further, research with third- and fourth-grade children in mathematics found that mismatches in children’s gestures and speech were related to types of strategies that adults used for instruction (Goldin-Meadow & Singer, 2003); in particular, adults offered instruction that contained more variability, with more different types of strategies, to children who produced mismatches than to children who did not. An open question for assessment then is how to use digital technology to track readiness to learn in a scalable manner.

#### Keep a record of the process of movements and get meaningful insight from it

Technology provides the opportunity for educators to keep track of student movements during the embodied learning process. For instance, certain types of movement patterns with digital manipulatives can illustrate that students have particular misconceptions. Further, being able to track students’ mathematical thinking process allows for different types of assessment questions that look at those processes, rather than simply their final answers, as part of learning assessments. For example, students could be asked to embody their schema of multiplication without physically indicating the operation directly. This could highlight schemas in a way that paper and pencil could not. A student may start small and move as if to get larger as a way to embody “multiplication makes things bigger.” And, if several different movements were recorded and students were to look at recordings of students acting out, they could be asked to classify which ones are embodying multiplication. This could provide insight about student cognition in a way that is missed without the merge of technology and movement.

## Conclusion

New technology offers many opportunities to improve instructional methods for embodied learning of mathematics. Throughout this process, it is crucial to not get carried away with the tool itself because it is not whether the tool is used or not, but rather the way the tool is being used, that impacts education. There are advantages to embodied learning and leveraging technology, such as the opportunities to track movements for assessment and to scale experiences that would be logistically difficult, for cost and space issues, to provide to students otherwise. However, it is important to remember that an embodied learning experience is not necessarily ideal for teaching all mathematical concepts to all students, but it might be desirable for some concepts and some students. It is up to future research to dissect those individual and contextual differences and to further discover how to integrate technology to improve the effectiveness of embodied learning for mathematics.

## Footnotes

1 Dehaene, Bossini, and Giraux (1993) were the first to describe the SNARC effect which describes a spatial-numerical association, probably reflecting how numbers are spatially oriented in one’s mind. In short, it was found that participants responded faster to smaller numbers with their left hand and also made fewer errors. With their right hand, they responded faster to larger numbers and also made fewer errors.
